# The Effect of LENA (Language ENvironment Analysis) for Children with Hearing Loss in Denmark including a Pilot Validation for the Danish Language

**DOI:** 10.3390/jcm13092688

**Published:** 2024-05-03

**Authors:** Jane Lignel Josvassen, Victoria Amalie Michael Hedegaard, Mie Lærkegård Jørgensen, Lone Percy-Smith

**Affiliations:** 1 Copenhagen Hearing and Balance Center, Rigshospitalet, Inge Lehmannsvej 8, Opgang 8, 3. Sal, 2100 Copenhagen, Denmark; victoria.amalie.michael.hedegaard@regionh.dk (V.A.M.H.); lone.percy.smith@regionh.dk (L.P.-S.); 2 Department of Health Technology, Hearing Systems Section, Computational Auditory Modeling, Technical University of Denmark (DTU), Ørsteds Plads, Bygning 352, 2800 Kongens Lyngby, Denmark; mielj@dtu.dk

**Keywords:** LENA (language environment analysis), LENA validation in Danish, AVT (auditory verbal therapy), early intervention, GDPR issues using LENA within the EU, children with hearing loss

## Abstract

**Background/Objectives:** This study aimed to investigate whether day-long recordings with Language Environment Analysis (LENA) can be utilized in a hospital-based Auditory Verbal Therapy (AVT) program in Denmark for children with hearing loss and to conduct a pilot validation in the Danish language. **Methods and materials:** A license for the LENA system (LENA SP) was purchased, and trials were offered to three families enrolled in the AVT program. Each family made two day-long recordings with 3–4 months in between and received feedback during the therapy sessions. From 18 × 10-min clips randomly pulled out of the recordings, a comparison of adult word counts (AWC) between the LENA algorithm counts and the counts made by two human transcribers was made and used for the pilot validation. **Results:** LENA proved to be valuable as a guiding tool for Danish parents. Pilot validation showed good correlations and an acceptable limit of agreement (LoA). **Conclusions:** LENA holds the potential for Danish validation and use in AVT/clinical practice. When used in clinical practice, parents must be informed of the biases and limitations, and possible ethical issues must be considered. Because of the GDPR rules, there is a need to discuss the possibility of implementing this tool clinically in Denmark and the EU.

## 1. Introduction

Neonatal hearing screening and knowledge of the importance of early intervention have resulted in a new generation of children with hearing loss (HL) who have better language outcomes [1,2,3]. Hearing is key to spoken language development, and children with HL develop spoken language in the same way as children with typical hearing. Even though hearing technology has developed significantly during the last decade, noisy environments and distance to the sound source are still challenging for children with HL, as they are exposed to less overhearing than their typically hearing peers. Overhearing accounts for a substantial proportion of spoken language learning [4]. Some children with HL can also have varying periods of hearing deprivation, for example, due to etiologies such as Pendred syndrome, which further delays spoken language development. Because of its high success rate [1], early intervention with auditory verbal therapy (AVT) is offered free of charge to parents of children with HL in Denmark in five different audiology departments [5]. In AVT, parents are guided and coached on how to support their child’s listening and spoken language development, and how to manage the consequences of less overhearing.

### Language Environmental Analysis (LENA)

LENA is a recording technology that allows parents, researchers, and clinicians to gain insight into children’s everyday listening and spoken language environments. The LENA software was developed with the primary goal of generating simple, high-level feedback on a child’s natural language environment to promote adult behavioral changes [6]. The software uses an algorithm that estimates the number of words spoken based on specific information in the speech signal, such as syllable count, consonant distribution, and segment duration. The LENA system has been validated in English for children up to 48 months [6].

Through day-long recordings that were obtained by a small wearable device (digital language processor (DLP)) and processed by the LENA software, it is possible to assess factors related to spoken language; that is, the software algorithm counts the number of adult words (adult word count—AWC), conversational turns (conversational turn count—CTC), and child vocalizations (child vocalization count—CVC). The LENA software generates a report that also provides an overview of the presence of noise, silence, overlapping speech, distant speech, TV/computer sound, and thus the quantity of meaningful language input throughout the day. All of these elements are important factors to consider in relation to listening and spoken language learning. 

The LENA report provides a visual timeline for the day and the presence of these elements, which is especially helpful in clinical contexts. Such, and even more detailed information from the LENA-developed software Advanced Data Extraction (ADEX) is being used by researchers worldwide studying spoken language learning in different contexts and programs for parents who want to support their child’s language development [7,8,9]. In several studies [10,11,12], LENA has also been shown to be a valuable objective tool for guiding parents of children with HL. LENA can assist families in creating favorable surroundings, increasing language stimulation, and decreasing the presence of noise. All of these help counter the negative effects of less overhearing and help children develop their listening skills and spoken language. 

The LENA system has the potential to be used as an integral part of AVT practice in Denmark, and this study investigates the feasibility for caregiver uptake, parent/child acceptance of using the DLP, the transferability of the results in everyday life, and hence its value for Danish parents of children with HL participating in AVT. 

This study also includes a pilot validation in the Danish language, which comprises LENA counts versus selections of audio recordings transcribed and counted by two native Danish-speaking listeners. Owing to time constraints, AWC was the primary focus of this study. AWC is commonly used in validity studies because of its precise transcription opportunities. Furthermore, AWC is recommended by the LENA Foundation along with CTC for validation purposes [13].

This study was funded by Interfond and conducted at the Copenhagen Hearing and Balance Center, Rigshospitalet, Denmark.

## 2. Materials and Methods

### 2.1. Participants

Three native Danish-speaking families with children with HL participated in this study. All of the children had undergone newborn hearing screening and had bilateral hearing loss. Two of the children wore bilateral hearing aids (HA), and one had bilateral cochlear implants (CI). The ages of the three participating children at the first recording were as follows: child 1, 35 months; child 2, 29 months; and child 3, 41 months. None of the children were diagnosed with other disabilities. All families were enrolled in AVT at the Copenhagen Hearing and Balance Center, Department of Otorhinolaryngology, Head and Neck Surgery and Audiology, Rigshospitalet, and were recruited through the AVT program. The families attended AVT sessions biweekly or monthly. All participants received written information about the study and provided written consent for the transfer of audio data to a country outside the EU (USA). 

### 2.2. Recording Procedure and Data Collection

All families received a DLP/LENA recorder, clothing to wear with the recorder, and the log form developed for this study. The families made two day-long recordings with 3–4 months in between. Families handed in recorders with stored recordings after the first recording. The recorders were handed out for a second recording.

The families were given instructions on how to carry out day-long recordings at home. Families were asked to avoid recording on a day with longer social visits, such as birthday parties. If a family spent time with other family members not living in the household or with non-family members, they were asked to inform them about the recording to avoid third-person recordings without consent [14] and to make a note in their log form about it.

The log form developed for this study consisted of a timetable with a space for noting events, people present, or shifts during the day. The log form was used for comparison with the LENA software-generated timeline.

Families invested time in using the DLP/LENA recorder at home. Guidance and feedback were provided during the AVT sessions. To respect the participants’ autonomy and give them control, the parents could make a note in the log form about specific passages during the day/recording that they did not want researchers to listen to [14]. Families could call for help during the recording day if they experienced problems handling the recording device.

The recordings were required to be at least 10 h long, as the LENA system requires 10 h long recordings for automatic analysis and percentile ranking [13]. All recordings received were 16 h long.

The data in the LENA reports were derived in comparison with normative data for typically developing American children with typical hearing [6,15,16].

As the LENA server is placed in Oregon, USA, the study required data transfer to a country outside the EU. Risk evaluation was performed by the Data Protection Agency, and dispensation from the Board of Management at Rigshospitalet was obtained for this study.

This study was conducted in accordance with “The Danish Code of Conduct for Research Integrity”. Participation in the study was voluntary and informed and written consent was obtained. Families were able to withdraw their consent and stop participating in the study without further notice.

### 2.3. Transcription Procedure for Pilot Validation

As previously mentioned, the LENA system is based on algorithmic data and has multiple parts that can introduce errors. Thus, the system must be validated for individual languages [13,17]. LENA has been validated in several languages, such as English, Spanish, European French, Dutch, Swedish, Vietnamese, and Korean [8,18], but not in Danish. A pilot validation in the Danish language was conducted. The LENA algorithm is developed in English, but because the Danish language is comparable to English in many aspects, major difficulties for the LENA algorithm to handle Danish were not expected.

Different validation studies have used varying transcription procedures and counts, but most studies have included adult word counts (AWC) [8,18]. Conversational turn count (CTC) and child vocalization count (CVC) are important measures but are not included as often as AWC [8,18].

As mentioned, AWC was the primary focus of this study. Three 10-min segments of each of the six recordings were selected, representing periods of high (top 10%), medium (middle 20%), and low (bottom 10%) interaction, as recommended by LENA for validation purposes [13]. It is important to use this approach because varying levels of speech activity help obtain a realistic distribution of typical events and errors [6]. The first 10 min of each recording was avoided because it may have been influenced by parental habituation to the idea of being recorded.

Numeric data resulting from the algorithmic analyses of the LENA SP software were extracted from the XML-formatted LENA ITS files via the LENA-developed program Advanced Data Extraction Tool (ADEX) version 1.1.3-5 r 10725 and processed with a LENA-developed syntax “SPSStop10mid20bottom10 script” (for AWC) in SPSS. The syntax randomly pulled out the three periods of interaction mentioned above. The time data from SPSS were transformed into a .txt file which, through the Audacity software version 3.1.3, allowed for the extraction of the three 10-mim segments in the locally stored .waw file with the day-long recording.

In total, 18 × 10-min segments or 180 min were pulled out from the six recordings for human transcription and statistical analyses.

Two native Danish-speaking transcribers (one speech and language pathologist (SLP) and one SLP student) listened to and transcribed the segments. Before the transcriptions, both transcribers (Transcriber 1 (T1) and Transcriber 2 (T2)) were familiarized with the LENA transcription guide, a set of counting rules recommended by the LENA Foundation [13], which was adapted to the Danish language, with Danish examples provided by the author.

### 2.4. Statistical Analyses

Statistical analyses were conducted using RStudio (Posit Team, 2023), R v. 4.3.1 (R Core Team, 2023), and the tidyverse v. 1.3.0 [19], ggplot2 v. 3.4.4 [20], and smplot2 v. 0.1.0 [21] packages. Pearson correlations were used to correlate the manual transcription counts T1 and T2 and to correlate the manual word counts with the LENA estimates. Furthermore, the manual and LENA word counts were compared using the Wilcoxon signed rank test. Bland–Altman analyses were used to investigate the agreement between the manual word counts and the LENA estimates. The average difference between the two methods represents the bias, while the limit of agreement (LoA = bias ± 1.96 × SD) is the interval that contains 95% of the differences observed between the two methods. The fixed bias was tested with a one-sample Wilcoxon signed rank test, while the proportional bias was tested with a linear regression.

### 2.5. Feedback to Parents

After each recording, parents received face-to-face feedback based on the results of the LENA report. The log form filled in by the parents during the day of recording was also considered and compared with the LENA report during feedback. After the final recording and feedback, a brief questionnaire was administered.

### 2.6. Questionnaire 

Families completed a brief questionnaire on the use of LENA in their homes. The questionnaire consisted of six closed-ended questions, and answers were provided through a closed set of five statements on a Likert scale, with a neutral option in the middle.

Example:

1. How did you find handling and using the LENA recorder?
**Very hard****Hard****Neutral****Easy****Very Easy**     

Furthermore, the parents were asked the following questions:

2. Did the fact that your child was wearing the recorder change your typical manner of interacting with your child? To a large extent, to some extent, neutral, not much, not at all.
3. How did you find being recorded at home? Very unpleasant, a little unpleasant, neutral, fine, very fine.
4. Did you experience challenges in making your child wear the recorder? To a large extent, to some extent, neutral, not much, not at all.
5. Did the feedback result in the implementation of new strategies in the home environment according to the feedback received? Not at all, not much, neutral, to some extent, to a large extent.
6. Would you recommend LENA as a guiding tool for other parents participating in AVT? Not at all, not much, neutral, to some extent, to a large extent.

The parents also had the option to write further comments.

## 3. Results

### 3.1. Reports and Feedback to Parents

After each recording, a LENA report was generated at https://auth.o.lena.org (accessed on 10 April 2024) for each child. Feedback from the results was provided during the subsequent AVT session. Parents were informed that the data generated by the LENA software might be biased because of the missing Danish validation and that LENA counts in general are an estimate and not a precise measurement [8,22].

Parents were informed that the AWC did not provide any information about the quality of speech, only the number of words spoken near the child, and that the report provided no information about whether the speech was child-directed. Research has shown that the number of conversational turns is a very important factor in language development [22,23,24] The guidance given to the parents emphasized the CTC, mindful of the fact that there is no information about the quality of the CTC from the report. Furthermore, the parents received information about the CVC, which is related to the CTC and the child’s verbalizations in daily living and play, and as such, relates to the child’s expressive language development. As with the AWC and CTC, the parents were informed that they would not obtain any information about the quality of the verbalizations from the report alone.

In both recordings, the children had an AWC within the 50–74 percentile, which corresponds to high average, or the 75–99 percentile, which corresponds to high. The percentile rankings were based on American norms [15,16]. Children 1 and 2 had a higher AWC in the second recording, and child 3 had a slightly lower AWC (Table 1, first row).

In both recordings, the children had a CTC within the 50–74 percentile, which corresponds to high average, or the 75–99 percentile, which corresponds to high (Table 1, second row). 

In both recordings, the children had a CVC within the 50–74 percentile, which corresponds to high average, or the 75–99 percentile, which corresponds to high, except child 1, who in the second recording had a CVC within the 25–49 percentile, which corresponds to low average (Table 1, third row).

### 3.2. Individual Considerations from Feedback and Guidance to the Parents

Child 1: Child 1 had a large drop in the CTC from the first to the second recording (Table 1; first column, second row) and a similar drop in the CVC (Table 1; first column, third row). In general, child 1 had very high counts for all measures in the first recording. From the log form and a discussion with the mother, it was documented that the day of the first recording was a day with family time in the home with both parents and an older sibling at home. Mother and child 1 also engaged in some activities, the two of them being together during the day. The results from the first recording were ideal and, presented with the results, the mother expressed that knowing what that day had been like would serve as a goal for other days. On the second recording day, the family spent hours with friends outside the home. The AWC increased, but as mentioned, the CTC and CVC decreased, which draws attention to the fact that the AWC cannot stand alone when it comes to explaining the factors that impact children’s language development. 

When presented with the data from the second recording, the mother expressed that it was an eye-opener to see the difference between the two days. Social life outside the small family circle should, of course, not be avoided, but as the mother expressed, the results drew her attention toward child 1′s possibilities for more active involvement in such situations. 

The above findings are also reflected in the report on audio environments (Table 2, first column). From the first to the second recordings, there was more distant speech, more overlapping speech, and less meaningful speech. In general, there was a low number of minutes of noise and TV/electronic sounds in both recordings, which is preferable for children with HL.

Child 2: On both recording days, child 2, the parents, and an older sibling were in the home together. Discussing the results from the first recording, the parents stated that they would increase their focus on the number of CTC during the day. All counts increased on the second recording, which did not surprise the parents, as they had made changes in their communication approach at home (Table 1, second column). 

The above findings were also reflected in the report on audio environments (Table 2, second column). From the first to the second recordings, there was less distant speech, less overlapping speech, and more meaningful speech. In general, there was a low number of minutes in noise and TV/electronic sound for child 2 in both recordings.

Child 3: On both recording days, the family was at home but also outside the home, visiting other family members. The results from the first recording showed high counts for all measures, and the discussion focused on different types of counts related to language development. The parents stated that they would increase their focus on CTCs. In the second recording, the CTC and CVC increased slightly (Table 1; third column, second and third row), but the AWC decreased slightly (Table 1; third column, first row). 

The findings are also reflected in the report on audio environments (Table 2, third column). From the first to the second recording, there was less distant speech. Simultaneously, slightly more overlapping speech and less meaningful speech were observed. In general, there was also a low number of minutes of noise for child 3 in both recordings but a slightly higher number of minutes with TV/electronic sound. The parents expressed that it had been helpful to see this report, and they felt more confident now when knowing more about what a day in their home looks like and how they can support their child’s language development. 

The results from the LENA reports corresponded well with the log forms and parental reports from the recording days. The feedback from the LENA report seemed accurate and useful for the parents who stated the learning outcome themselves and accordingly reflected on their communication practice and audio environments at home and, in some instances, made changes to support their child’s needs. 

The LENA report thus gives objective information that is well aligned with the strategies and goals of AVT. 

### 3.3. Questionnaire

Table 3 shows the results of the brief questionnaire administered to the three participating families after the second recording. Answers to the left on the Likert scale indicate negative experiences or unbeneficial ones, whereas those to the right indicate positive experiences and beneficial ones.

There was a box for further comments in question 5: *One family (child 1) wrote that “The consciousness of what a good day is like has been very important”. **One family (child 3) wrote that “We are now more aware of conversational turn-taking”.

There was also a box for further comments in general: ***one family (child 3) did not fill in question 6, but during conversation, they expressed that they would like to use LENA again and they wrote that it provides “Very good feedback that constructively helps us to optimize learning and development for XX”.

All answers, except for one (question 5), were to the right on the Likert scale, which strongly indicates that all three families had positive experiences and were profiting from using LENA.

### 3.4. LENA Reliability 

Agreement between the two manual transcribers, T1 and T2, was assessed on 18 segments pulled from the recordings of the 3 families that participated in the study. The average difference in word count between T1 and T2 was 6.1 words (SD = 18.0 words), and there was a strong positive correlation between the word counts of T1 and T2 (r(16) = 1.0; *p* < 0.001). 

Before comparing the manual and LENA word counts, possible outliers were identified. Outliers were defined as data points with a z-score larger than 2 standard deviations (SD) from the mean. One outlier was found in the LENA data and this observation was not included in the remaining part of the analysis. An analysis of the removed outlier can be found in Supplementary Figure S1. 

**Figure 1:**
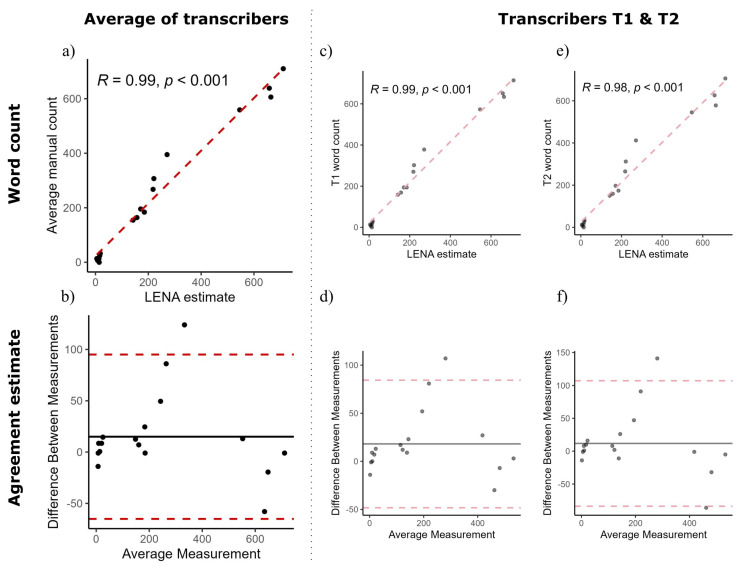
Correlations between manual word counts and LENA estimates (**top row**) and agreement estimates (**bottom row**). (**a**) Scatterplot of averaged manual word count from T1 and T2 and LENA estimate. (**b**) Bland–Altman plot of average transcribers’ word count x LENA estimate. (**c**) Scatterplot of T1 word count and LENA estimate. (**d**) Bland–Altman plot of T1 word count x LENA estimate. (**e**) Scatterplot of T2 word count and LENA estimate. (**f**) Bland–Altman plot of T2 word count x LENA estimate. R = correlation coefficient. Black line is mean of differences between two methods (bias), while red dotted lines are upper and lower 95% limits of agreement.

The average difference in word count between the manual and LENA counts was 14.9 words (SD = 39.6 words), and there was no statistically significant difference between the two types of word counts (V = 107; *p* = 0.16). Furthermore, there was a strong positive correlation between the manual counts and the LENA estimates (Figure 1a, r(15) = 0.99, *p* < 0.001). There were also strong positive correlations between each transcriber’s word count and the LENA estimates (Figure 1c,d). In comparison, an English validation found a correlation coefficient of r = 0.92 [25]. Other examples of correlations with AWC include a Swedish validation in which they found r = 0.67 [26], a French validation that found r = 0.64 [27], and a Dutch validation that found r = 0.87 [17].

Figure 1b shows the Bland–Altman plot for the agreement between the average word count of the two transcribers and the LENA estimate. The mean difference in estimating the bias between the two methods was 14.9 words, which was not significantly different from 0, and therefore, there was no fixed bias (V = 107, *p* = 0.16). Furthermore, a possible proportional bias that represents the variance of the differences across word counts was evaluated using linear regression. No proportional bias was found between the two methods (R2= −0.05; F(1,15) = 0.26; *p* = 0.62). Figure 1d,f show the Bland–Altman plots for the agreement between each transcriber’s word count and the LENA estimates.

## 4. Discussion

The questionnaire clarified that LENA is easy to understand and implement in daily life. The children accepted wearing the vest with the DLP/LENA recorder for a whole day and the parents found it acceptable to record at home. The questionnaire also showed that being recorded had some influence on the parents’ normal manner of interacting with their child, which is a potential bias in using LENA as a guiding tool. Two families found that they needed a little persuasion to get their child to wear the recorder in the vest in the morning. The vests in this study are all light blue. The parents expressed a wish for more colorful or cool patterns in the fabric, which they thought would promote immediate acceptance by their children. In a Canadian study from 2016 [10] investigating the use of LENA in an AVT setting, a similar questionnaire was administered to the participating five families and the answers were very similar to the answers reported in the present study.

The parents participating in this study accepted that the two investigators listened to 3 × 10 min from each recording. Listening to parts of the recording is necessary for validation purposes, and as soon as the validation is confirmed as eligible, recordings can only be digitally processed. The LENA system is used in different research areas and listening to recordings may be essential for answering the research question. When listening to all or part of the recordings, ethical issues must be considered. An important first step is to inform parents about the reason for listening, how much of the recording will be listened to, and by whom (researchers or clinicians). During a day at home, the exchange of sensitive and personal information can occur; therefore, it is important to give parents an option to note specific times during the day that they do not want investigators to listen to. GDPR (General Data Protection Regulation) rules, the risk of third-person recordings, and other ethical issues must always be considered when using LENA [14]. 

In Denmark, all children with HL are offered AVT. Occasionally, families where both parents are deaf and use Danish Sign Language choose to participate in AVT often with assistance from a hearing family member or the child’s pedagogue from daycare or kindergarten. It would not make much sense to offer LENA recordings in a home where Danish Sign Language is the primary language, as it is without sound. However, it would be interesting to analyze the children’s language environment in daycare or kindergarten, which in this case often is the primary environment for learning listening and spoken language. Data collected by the LENA Foundation [9] showed a significantly lower number of CTC in kindergartens than in the home environment. In addition to this, there is often a considerably noisier environment in kindergartens [28] than in a home environment. These are factors that are not preferable for any child and least preferable for a child with HL. Day-long recordings in daycare or kindergarten are generally interesting, as children in Denmark often spend many hours in daycare and kindergarten because it is common for both parents to work outside the home during hours in which children are awake and ready to learn. However, challenges must be expected in making LENA recordings in daycare or kindergartens in Denmark because of the GDPR and the risk of third-person recordings, but it is of interest to investigate. Professional guidance for creating optimal listening conditions for children with HL to help in their listening and spoken language development is pertinent in all situations involving children with HL.

The number of words per hour reported in Table 1 and the audio environment per hour reported in Table 2 are derived from the total number of hours recorded, which is 16 h. Thus, these numbers may be a little lower than what is found in other studies where only children’s waking hours are reported [8]. 

### 4.1. Pilot Validation

Validations of LENA in different languages showed differences in the correlation coefficients. This can, of course, be due to the way the LENA algorithm handles different languages, but also due to different counting methods. Some researchers have picked regions with high activity from the recording, while others have picked more diverse samples [17,18], as is the case in this study. Another explanation could be the individually applied counting rules from study to study [17]. In this study, the LENA transcription guide was adapted to the Danish language and used by the two transcribers. It can be argued that LENA’s own counting rules mimic the LENA algorithm and that using them will ensure good correlation and agreement. For example, the LENA transcription guide states that periods with noise and overlapping speech should not be included, even though they may be intelligible to an adult transcriber. This can be considered misleading. However, children are end-product users, and this adds up to children’s general need for a better signal-to-noise ratio (SNR) than adults [29], especially for this specific clinical group, children with HL, who need a better SNR than children with typical hearing [30]. Thus, ruling out periods with noise and overlapping speech provides a more realistic picture of what children with and without HL hear throughout the day.

Applying the Bland–Altman plot to the statistical analysis provides more knowledge of the agreement between the methods. The correlation coefficients indicate only whether there is a linear association between the two methods [31]. 

The average difference in word count between the manual and LENA counts was 14.9 words. The total LENA word count in the selected audio files was 4018 words. A total of 14.9 words is 0.37% of the total LENA word count. In the authors’ opinion, this difference seems acceptable both for research purposes and in clinical practice as long as you take the biases and limitations of the use of LENA into account and when parents are informed accordingly. 

Because there is no fixed threshold for when an agreement (LoA) is considered acceptable [31], it is up to Danish users of LENA to decide whether this agreement is acceptable. The LoA without the outlier (Figure 1) seems more acceptable than the LoA with the outlier (Supplementary Figure S1); however, the reality is that outliers/errors exist within the LENA system, which underlines the fact that the LENA system only provides an estimate of a child’s language environment and the need for professionals to be conscious of this fact when giving guidance to parents. For example, outliers can arise from the LENA algorithm wrongly labeling a child’s voice as a female voice, or vice versa [32].

Keeping in mind the biases inherent in this type of tool, the pilot validation showed a good correlation and, from the authors’ point of view, an acceptable agreement between methods for the AWC. 

The potential for actual Danish validation is presented. The LENA system has the potential to be used in different research areas that investigate children’s language development and language environments in Denmark and is advantageous when collecting large quantities of data [14,22].

Owing to time constraints, this study only comprises a pilot validation because of the small sample of recordings used (180 min) and the fact that only the AWC was used in the validation procedure. If Danish validation is carried out, it is preferable to look at LENA’s guidelines for validation that also include the CTC [13] and other researchers’ views on validation to determine the most suitable method; for example, including all types of counts, including both speech and non-speech classes for a more thorough evaluation as well as having a more diverse group of participants with regards to age and backgrounds. It might also be preferable to have more transcribers [17,32]. 

The parents were informed of the significance of each type of count in the LENA report. Hart and Risley (1995) [33] showed that the average number of words heard by a child has a direct impact on the child’s language development and academic success later in life. Hence, the AWC is interesting but cannot stand alone. Recent studies [23,24] have shown that conversational turns have a huge impact on language development, which is why the CTC and preferably CVC should also be included in the validation.

Furthermore, percentile rankings are based on the American norm [15,16], which does not necessarily align with the Danish norm, and this also needs to be considered.

### 4.2. Limitations of Study

This study was originally scheduled to run for 18 months (with one study day a week). For many years, LENA software has been purchased and installed on a local computer (LENA Pro), but this option is now being phased out. Instead, the processing of data is cloud-based (LENA SP). As the LENA server is placed in Oregon, USA, the data are sent to a country outside the EU, which does not align with EU practice and GDPR rules. This resulted in a prolonged approval procedure of 12 months, which resulted in large limitations to the original study design. Originally, this study included two recordings and two follow-ups separated by three months with six families. Owing to time constraints, only three families participated in this study. The plan was to transcribe the AWC, CTC, and CVC for validity measures, but only the AWC was transcribed. The approval for using cloud-based LENA software in this study was restricted to six patients and was not approved for use in clinical practice in general. If LENA is to be used as a research or clinical tool for parents in Denmark and other European countries, a solution must be found to comply with GDPR rules.

This study is limited by the small number of participants; however, this study shows that the LENA report gives objective information that is well aligned with the strategies and goals of AVT. The good results are encouraging and call for a new trial with more participants to investigate whether a larger group of parents will also benefit from using LENA as an objective tool to gain insight into everyday language stimulation and environmental factors. LENA recordings should always be optional for parents and should only be considered supplemental to the general guidance and coaching inherent in AVT.

## Figures and Tables

**Table 1:** LENA’s estimate of the number of adult word counts, conversational turns, and child vocalization counts for the three children throughout the recording day for the first and the second recording. The data are presented as the total amount per day (day column) and the rounded average amount per hour (hour column).

Count	Child 1				Child 2				Child 3			
	1st		2nd		1st		2nd		1st		2nd	
	Day	Hour	Day	Hour	Day	Hour	Day	Hour	Day	Hour	Day	Hour
Adult word count	17,877	1117	21,583	1349	13,437	840	15,955	997	14,343	896	14,021	876
Conversational turns	1359	85	552	35	568	36	740	46	872	55	968	61
Child vocalization count	6083	380	2098	131	2199	137	2607	163	4388	274	4764	298

**Table 2:** Audio environments from the day-long recordings analyzed by the LENA software in minutes per day. 1st are the recordings from the first day, while 2nd are the recordings from the second day. The data are presented as the total amount per day (day column) and the rounded average amount per hour (hour column).

Count	Child 1				Child 2				Child 3			
	1st (Min)		2nd (Min)		1st (Min)		2nd (Min)		1st (Min)		2nd (Min)	
	Day	Hour	Day	Hour	Day	Hour	Day	Hour	Day	Hour	Day	Hour
Silence/background	512	32	457	29	583	36	535	33	456	29	395	25
Noise	25	2	26	2	16	1	31	2	11	1	11	1
TV/electronic	12	1	58	4	9	1	11	1	44	3	81	5
Distant	71	4	88	6	92	6	81	5	107	7	94	6
Overlap	92	6	165	10	97	6	86	5	110	7	161	10
Meaningful	245	15	163	10	160	10	213	13	228	14	214	13

**Table 3:** Results from questionnaire.

Answer Options-Likert Scale	Very Hard	Hard	Neutral	Easy	Very Easy
Questions					
1. How did you find handling and using the LENA recorder?					3 answers
	to a large extent	to some extent	neutral	not much	not at all
2. Did the fact that your child was wearing the recorder change your typical way of interacting with your child?				3 answers	
	very unpleasant	a little unpleasant	neutral	fine	very fine
3. How did you find being recorded at home?					3 answers
	to a large extent	to some extent	neutral	not much	not at all
4. Did you experience challenges making your child wear the recorder?				2 answers	1 answer
	not at all	not much	neutral	to some extent	to a large extent
5. Did the feedback result in the implementation of new strategies in the home environment according to the feedback received?		* 1 answer		** 1 answer	1 answer
	not at all	not much	neutral	to some extent	to a large extent
6. Would you recommend LENA as a guiding tool for other parents in AVT?					*** 2 answers

## Data Availability

The datasets generated and/or analyzed during the current study are available from the corresponding author upon request.

## Supplementary Materials

The following supporting information can be downloaded at: https://www.mdpi.com/article/10.3390/jcm13092688/s1, Figure S1. Correlations between manual word counts and LENA estimates (top row) and agreement estimates (bottom row) including all measured data points. (a) Scatterplot of averaged manual word count from T1 and T2 and LENA estimate. (b) Bland-Altman plot of the average transcribers’ word count x LENA estimate (c) Scatterplot of T1 word count and LENA estimate. (d) Bland-Altman plot of T1 word count x LENA estimate (e) Scatterplot of T2 word count and LENA estimate. (f) Bland-Altman plot of T2 word count x LENA estimate. R = correlation coefficient. The black line is the mean of the differences between the two methods (bias), while the red dotted lines are the upper and lower 95% limits of agreement.
