# Supplementary material for: The Effect of LENA (Language ENvironment Analysis) for Children with Hearing Loss in Denmark including a Pilot Validation for the Danish Language
Source: J Clin Med. 2024 May 3;13(9):2688. doi: 10.3390/jcm13092688 (PMC11084579; doi:10.3390/jcm13092688)
Supplement: Supplementary file 1 [file jcm-13-02688-s001.zip › jcm-2982420-supplementary.pdf]

Journal of
Clinical Medicine

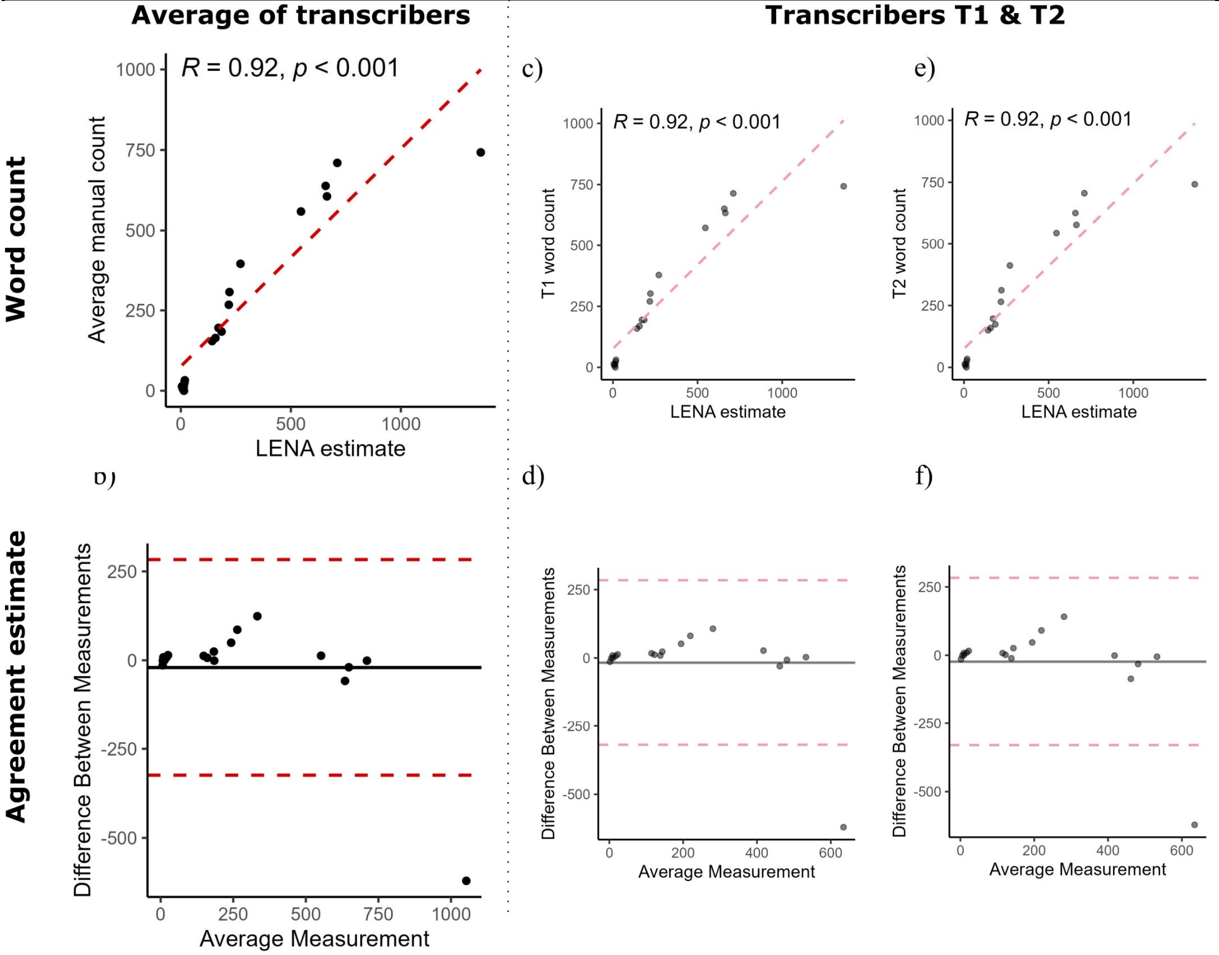

Supplementary Figure S1. Correlations between manual word counts and LENA estimates (top row) and agreement estimates (bottom row) including all measured data points. a) Scatterplot of averaged manual word count from T1 and T2 and LENA estimate. b) Bland-Altman plot of the average transcribers' word count x LENA estimate c) Scatterplot of T1 word count and LENA estimate. d) Bland-Altman plot of T1 word count x LENA estimate e) Scatterplot of T2 word count and LENA estimate. f) Bland-Altman plot of T2 word count x LENA estimate. R = correlation coefficient. The black line is the mean of the differences between the two methods (bias), while the red dotted lines are the upper and lower 95% limits of agreement.
